# Radiation induced apoptosis and initial DNA damage are inversely related in locally advanced breast cancer patients

**DOI:** 10.1186/1748-717X-5-85

**Published:** 2010-09-24

**Authors:** Beatriz Pinar, Luis Alberto Henríquez-Hernández, Pedro C Lara, Elisa Bordon, Carlos Rodriguez-Gallego, Marta Lloret, Maria Isabel Nuñez, Mariano Ruiz De Almodovar

**Affiliations:** 1 Radiation Oncology Department, Hospital Universitario de Gran Canaria Dr. Negrín, Spain; 2 Instituto Canario de Investigación del Cáncer (ICIC), Spain; 3 Clinical Sciences Department, Universidad de Las Palmas de Gran Canaria, Spain; 4 Immunology Department, Hospital Universitario de Gran Canaria Dr. Negrín, Spain; 5 Radiology Department, Hospital Universitario San Cecilio, Universidad de Granada, Spain

## Abstract

**Background:**

DNA-damage assays, quantifying the initial number of DNA double-strand breaks induced by radiation, have been proposed as a predictive test for radiation-induced toxicity. Determination of radiation-induced apoptosis in peripheral blood lymphocytes by flow cytometry analysis has also been proposed as an approach for predicting normal tissue responses following radiotherapy. The aim of the present study was to explore the association between initial DNA damage, estimated by the number of double-strand breaks induced by a given radiation dose, and the radio-induced apoptosis rates observed.

**Methods:**

Peripheral blood lymphocytes were taken from 26 consecutive patients with locally advanced breast carcinoma. Radiosensitivity of lymphocytes was quantified as the initial number of DNA double-strand breaks induced per Gy and per DNA unit (200 Mbp). Radio-induced apoptosis at 1, 2 and 8 Gy was measured by flow cytometry using annexin V/propidium iodide.

**Results:**

Radiation-induced apoptosis increased in order to radiation dose and data fitted to a semi logarithmic mathematical model. A positive correlation was found among radio-induced apoptosis values at different radiation doses: 1, 2 and 8 Gy (p < 0.0001 in all cases). Mean DSB/Gy/DNA unit obtained was 1.70 ± 0.83 (range 0.63-4.08; median, 1.46). A statistically significant inverse correlation was found between initial damage to DNA and radio-induced apoptosis at 1 Gy (p = 0.034). A trend toward 2 Gy (p = 0.057) and 8 Gy (p = 0.067) was observed after 24 hours of incubation.

**Conclusions:**

An inverse association was observed for the first time between these variables, both considered as predictive factors to radiation toxicity.

## Background

Radiation induced normal tissue damage is the most important limitation for the delivery of a high potentially curative radiation dose. Radiation doses are limited by the tolerance of normal tissues included in the treatment volume. Intrinsic variations in radiosensitivity determine most of the individual differences in normal tissue damage [1-3]. It is possible to determine the individual radiosensitivity before radiotherapy (RT) [4,5]. If intrinsic differences in individual radiosensitivity were responsible for the variation in severity of early or late radio-induced toxicity [6-9], we could adjust the radiation dose to be delivered. An association between DNA-damage assays, quantifying the initial number of DNA double-strand breaks (DSB) induced by radiation, and radiation-toxicity has been reported [10,11]. Increasing numbers of radiation induced DSB were related to severe late toxicity in breast cancer patients [10]. Determination of radiation-induced apoptosis (RIA) in peripheral blood lymphocytes (PBLs) by flow cytometry analysis has been proposed as a possible prediction value of normal tissue responses after RT [12,13]. RIA was predictive of late toxicity in several tumour locations [14-16]. Patients suffering of late toxicity after RT showed reduced rates of RIA. Furthermore, patients affected by the Ataxia-Telangiectasia (AT) syndrome showed the lowest rates of RIA. Defective apoptotic response to radiation in the PBLs of those sensitive patients could help to explain this association. As described above, late toxicity in breast cancer patients treated with radiation therapy has been related to increased radiosensitivity of lymphocytes, as shown by increased number of DSB/Gy/DNA unit, and reduced RIA. According to this, sensitive patients would show increased number of DSB and reduced RIA as results of defective apoptotic processing of the initial damage induced by x-rays. Considering the above background and observations, the aim of the present study was to analyze if there was any statistical relation between initial DNA damage, estimated by the number of DSB, and the apoptotic rates observed estimated by the amount of RIA.

## Methods

### Patients

PBLs were taken from 26 consecutive patients with locally advanced breast carcinoma (stage IIIa-IIIb), diagnosed and treated in our institution, and given inform consent. All patients were referred to receive high-dose hyperfractionated radical radiotherapy as follows: 60 Gy to the whole breast over a period of 5 weeks in two daily fractions of 1.2 Gy separated by at least 6 h on 5 days each week, and followed by a boost of 21.6 Gy to a total dose of 81.6 Gy. The study was approved by the Research & Ethics Committee of our institution. Mean age of patients was 57.62 ± 12.99 years (range 30-83), 69.2% of them were menopause women.

### Apoptosis assay and flow cytometry

RIA analyses were performed as previously reported [13,17]. PBLs were irradiated with 0, 1, 2 and 8 Gy. After irradiation, samples were incubated for 24 hours at 37°C and 5% CO_2_. After extraction of cellular pellet, it was resuspended in 100 μl Annexin V buffer Kit (Pharmingen, Becton Dickinson). After the addition of 4 μl of Annexin-V-FITC and 10 μl of propidium iodure (PI), cells were incubated during 15 minutes at room temperature in the dark. Finally, 400 μl of Annexin V buffer Kit were added. Every assay was made in triplicate. The flow cytometry analysis was performed in a FACScalibur (Becton Dickinson, San José, CA) using a 488 nm argon laser. Each sample was analyzed using 5000 events/sample acquired in list mode by a Macintosh Quadra 650 minicomputer (Apple computer Inc., Cupertino, CA). Data were analyzed using the CellQuest program (Becton Dickinson, San José, CA) calculating early and late apoptosis levels. RIA is defined as the percentage of total PBLs death induced by the radiation dose minus the spontaneous cell death (control, 0 Gy).

### DNA damage assay

Data related to initial DNA damage were obtained from our files [10]. Shortly, mononuclear cells were isolated from blood of patients, resuspended in cold DMEM, and mixed with 1% ultra-low-melting-point agarose to obtain 250 μl plugs. Irradiation on ice was performed using a ^60^Co source (rate dose 1.5 Gy/min, approximately) as previously reported [10]. Plugs were held 1 hour at 4°C and incubated at 37°C for 24 hours. The study of initial DNA damage was completed in the University of Granada (Spain). Initial radiation-induced DNA damage in PBLs was measured as previously described [18] and was considered an individual indicator of the molecular radiosensitivity of normal cells.

### Statistical analyses

Statistical analyses were performed using the SPSS Statistical Package (version 15.0 for Windows) as previously reported [10,13,18]. The cut-off value for DSB/Gy/DNA unit was the median. Additional cut-off values studied were the tertiles of the distribution. All tests were two sided and statistical significance level was established for a p value less than 0.05.

## Results

### Radiation-induced apoptosis

**Figure 1:**
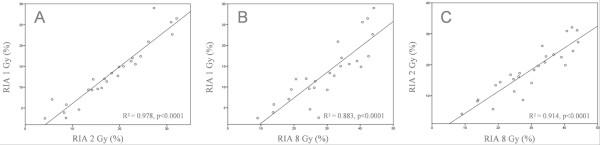
**Correlation between radio-induced apoptosis data at the different doses of radiation**. Panel A: 1 vs. 2 (Gy); Panel B, 1 vs. 8 (Gy); Panel C: 2 vs. 8 (Gy). A linear correlation was established.

Data of RIA were available in all 26 breast cancer patients, as shown in Table 1. RIA values increased with radiation dose (Table 1), and data fitted to a semi-logarithmic equation as follows: RIA = α + β ln(Gy), confirming our previously observations [13,17,19]. The increments in RIA were defined by two constants: the coefficient in origin α (as the origin of the curve in the Y axis determining the spontaneous apoptosis); and the coefficient β defining the slope of the curve. α and β followed a normal distribution (Kolmogorov-Smirnov test, p > 0.05). Mean of β was 7.93 ± 2.68 standard deviation (range 1.64-26.63; median, 12.64); mean of β was 7.93 ± 2.68 (range 3.18-12.57; median, 7.85) (Table 1). In this way, we were able to establish an individual radiosensitivity value defined by two constants: α as the spontaneous apoptotic rate, and β as the percentage of RIA per Gy. A good correlation was found among RIA data at different doses: 1 vs. 2 Gy, R^2 ^= 0.978 (p < 0.0001); 1 vs. 8 Gy, R^2 ^= 0.883 (p < 0.0001); 2 vs. 8 Gy, R^2 ^= 0.914 (p < 0.0001) (Spearman Rho test, Figure 1). The experimental data showed an excellent fit to the described model (median regression coefficient at 24 h was 99.58).

**Table 1:** Apoptosis data obtained after the irradiation of PBLs at 1, 2 and 8 Gy

Absolute data	Mean ± SD	Median (range)
**RIA 1 Gy**	13.33 ± 7.26	12.36 (2.51-29.00)
**RIA 2 Gy**	18.20 ± 7.82	17.79 (4.17-32.08)
**RIA 8 Gy**	29.70 ± 10.05	30.44 (9.02-44.10)
**DNA-DSB**	1.70 ± 0.83	1.46 (0.63-4.08)

**Defined model data**		

**α Coefficient**	13.08 ± 7.21	12.64 (1.64-26.63)
**β Coefficient**	7.93 ± 2.68	7.85 (3.18-12.57)
**Regression coefficient**	98.18 ± 4.58	99.58 (82.49-100)

### Relation to initial DNA damage

**Figure 2:**
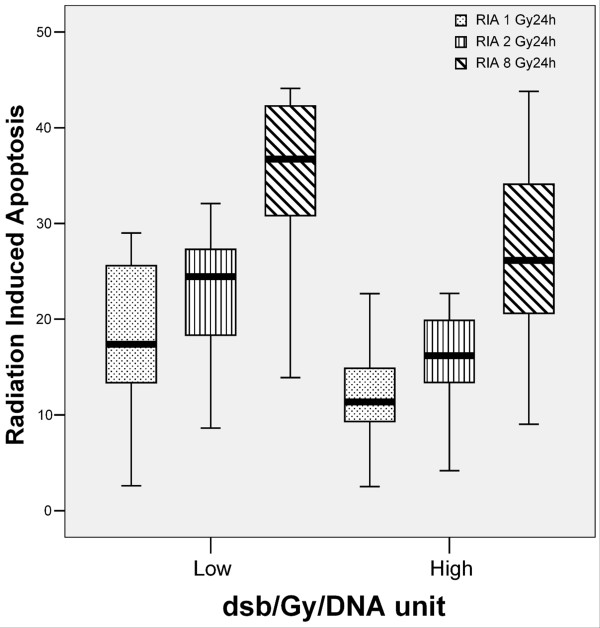
**Box plot shows an association between DSB and RIA**. The lines connect the medians, the boxes cover the 25th to 75th percentiles, and the minimal and maximal values are shown by the ends of the bars. Patients with lower amount of DSB suffered higher levels of RIA.

Mean ± standard deviation of DSB/Gy/DNA unit, obtained from our files [10] was 1.70 ± 0.83 (range 0.63-4.08; median, 1.46). No relation was found between the number of DSB and the RIA at 1 (p = 0.406), 2 (p = 0.592) and 8 Gy (p = 0.619). In the same way, no relation was found between the number of DSB and the model coefficient variables α (p = 0.457) and β (p = 0.901), when they were analyzed as continuous variables (Pearson test used in all correlations). When DSB values were segregated in two groups (the lower third against the two upper thirds of the distribution), a modest inverse correlation was found, reaching statistical significance for RIA at 1 Gy (p = 0.034). A similar trend was found for RIA at 2 (p = 0.057) and 8 Gy (p = 0.067) (Figure 2). α values also showed an inverse correlation with DSB, that reached statistical significance (p = 0.041). No relation was found between the number of DSB and β constant.

## Discussion

We established for the first time, a statistical association between initial DNA damage, measured as DSB, and RIA. Ionizing radiation (IR) kills cells by damaging the structure and function of genomic DNA. The response of cells to this damage and their ability to restore DNA sequence integrity remains unclear. Intrinsic radiosensitivity is correlated in a first approach to the ability of the cell to detect and repair DNA damages. DSB can be induced by a variety of DNA damaging agents, such as x-rays [20]. Differences in cell survival may be related to the number of initial DNA DSB, the DSB rejoining rate, or the level of residual DNA damage [11,21-24]. Wide variation in the level of initial radiation-induced DNA damage suggests that variation in cell radiosensitivity can be detected in vitro using radiosensitivity assays on PBLs from normal tissues of cancer patients prior to RT [11]. Patients with radiosensitive PBLs presented a significant increased risk for develop late complications [25]. Increasing numbers of radiation induced DSB were related with severe late toxicity reactions in breast cancer patients [10]. In the other hand, RIA values, that fitted to a semi logarithmic model defined by α and β constants, increased with radiation dose [13]. Previously studies were uniformly positive towards a relation between RIA and radiation toxicity [14]. In fact, patients suffering of late toxicity after RT showed reduced levels of RIA. The mechanism behind the relationship between increased radiation toxicity and reduced apoptotic response in PBLs is still unclear. Thus, lymphocytes from patients who suffered different syndromes related with radiosensitivity (i.e., Ataxia-telangiectasia, Bloom syndrome, or Fanconi anaemia) showed absence of induction of p53 [26,27] and lower levels of Bax [28]. This failure in the induction of the apoptosis response in lymphocytes has been related with late toxicity [16]. So, defective apoptotic response to radiation in PBLs could help to explain this inverse relation [14]. We report here for the first time a statistical association between these two predictive values for radiation toxicity. Lowest values of initial DNA damage were related to higher values of RIA, at the same radiation dose. This relation was also observed between DSB and the spontaneous apoptosis of cells (estimated by the α constant). Cell response to x-rays is individual, and the amount of initial DNA damage depends on each patient. The two main mechanisms of DSB repair are 1) non-homologous end joining (NHEJ) and homologous recombination repair (HRR) [29,30], and 2) cell-cycle checkpoints that provide time for repair and apoptosis [31]. Depending on the severity of the DNA damage, cells may undergo apoptosis instead of attempting to repair the damage [32]. Regulation of RIA and cell cycle arrests is achieved primarily through p53 phosphorylation by ATM protein [33]. T-lymphocytes from AT patients display severely compromised apoptotic response, as well as non-induction of p53 after exposure to IR [26]. Moreover, PBLs from AT patients are characterized by an elevated spontaneous level of apoptotic cells compared to normal ones [26]. Extremely radiosensitive patients have abnormalities in their ability to recognize or repair the DNA DSB typically induced by IR [15]. Gene expression profile of irradiated PBLs showed that the majority of the strongly activated genes were p53 targets involved in DNA repair and apoptosis [28]. The level of BAX activation correlated with the sensitivity of the cells to radiation [28]. A link between RIA and cellular response to DNA DSB arises because there are many proteins common to the execution of both processes. Anyhow, there are yet unidentified proteins or complexes that regulate the cross-talk between the mutually exclusive pathways of maintenance of life and initiation of death [31]. How these pathways are integrated to provide a concerted response to DSB is very complex, and could help to understand the inverse relation between the initial DNA damage to IR and RIA.

## Conclusion

A statistical inverse association was observed for the first time between DNA-DSB and RIA in 26 patients diagnosed with locally advanced breast cancer. However, these results must be verified in larger series of patients.

## List of abbreviations

AT: Ataxia-Telangiectasia; DSB: Double-strand Break; PBLS: Peripheral Blood Lymphocytes; PI: Propidium Iodide; RIA: Radio-induced Apoptosis; RT: Radiotherapy.

## Competing interests

The authors report no conflicts of interest. The authors alone are responsible for the content and writing of the paper.

## Authors' contributions

BP has been involved in conception and design of the project and have made the selection of patients, the evaluation of clinical variables and grade of toxicity as well as all the aspects related with the patients selected, including the treatment. LAHH has written the manuscript, has made tables and figures and has been involved in type of packaging likewise in the submission process. PCL has been involved in conception and design of the study and in drafting the manuscript and has given final approval of the version to be published. EB and CRG have made the cell experiments with lymphocytes, irradiation of cells, flow cytometry experiments, data acquisition and statistical analysis. ML has made the selection of patients, the evaluation of clinical variables and grade of toxicity as well as all the aspects related with the patients selected, including the treatment. MIN and MRDA have been involved in conception and design of the study, in drafting the manuscript, and have made the DNA-DSB experiments and analyses. All authors read and approved the final manuscript.
